# Potential Benefit of Structural Health Monitoring System on Civil Jet Aircraft

**DOI:** 10.3390/s22197316

**Published:** 2022-09-27

**Authors:** Vincenzo Cusati, Salvatore Corcione, Vittorio Memmolo

**Affiliations:** 1 Design of Aircraft and Flight Technologies Research Group, Department of Industrial Engineering, University of Naples Federico II, 80125 Napoli, Italy; 2 Aerospace Structures and Materials Laboratory, Department of Industrial Engineering, University of Naples Federico II, 80125 Napoli, Italy

**Keywords:** damage detection, aircraft structures, cost-benefit analysis, implementation strategies, multi-disciplinary analysis, direct operating cost

## Abstract

Structural health monitoring represents an interesting enabling technology towards increasing aviation safety and reducing operating costs by unlocking novel maintenance approaches and procedures. However, the benefits of such a technology are limited to maintenance costs reductions by cutting or even eliminating some maintenance scheduled checks. The key limitation to move a step further in exploiting structural health monitoring technology is represented by the regulation imposed in sizing aircraft composite structures. A safety margin of 2.0 is usually applied to estimate the ultimate loading that composite structures must withstand. This limitation is imposed since physical nondestructive inspection of composite structures is really challenging or even impossible in some cases. However, a structural health monitoring system represents a viable way for a real time check for the health status of a composite structure. Thus, the introduction of structural health monitoring should help into reducing the stringent safety margin imposed by aviation regulation for a safe design of composite structures. By assuming a safety margin reduction from 2.0 to 1.75 thanks to the installation of permanently attached sensors for structural health diagnostics, this paper assesses the potential fuel savings and direct operating costs through a multidisciplinary analysis on a A220-like aircraft. According to the foreseen level of technology, addressed through the number of sensors per square meter, a DOC saving from 2% up to 5% is achievable preserving, at the same time, all the key aircraft performance.

## 1. Introduction

Structural Health Monitoring (SHM) is mainly devoted to continuously inspect maintenance critical components posing new perspective in designing lighter, safer, and eventually cleaner aircraft. In principle, SHM is expected to avoid or reduce typical accommodations employed during design (e.g., composite knockdown factors) and lifetime management (strict scheduled inspection), inducing a cost-effective maintenance [1]. Due to the promising impact on aircraft operation management, several approaches have been conceived and advanced in the last decades, including a variety of techniques and technologies. Among them, the use of permanently installed sensors on the airframe or embedded within it, raised the attention of the scientific community as it can provide both global and local feedback about the structural health. In principle, this approach facilitates on-demand in situ measurements enabling a sort of trend monitoring in the observed system behavior [2]. The use of time or frequency domain data all over the lifetime can indeed provide an overview of anomalies, track their position and severity. In addition to that, starting from diagnosis carried out, it is possible to estimate residual useful life of the monitored object and eventually extend the inspection intervals [3]. As to the diagnosis phase, several techniques have been focused by the researchers to be applied to aircraft, including ultrasonic guided waves [4,5], guided electromagnetic waves [6], electromechanical impedance [7], and vibration response [8]. Generally, this approach can be either passive (e.g., activated by aircraft boundary layer [9]) or active [10] (i.e., enabled by the system). In every case, a damage feature based on characteristics of measurement data is adopted as to be sensitive to the effect of defect on the properties of the observed structure. Hence, the damage indicator directly correlates measurement from a cluster of sensors with the presence and extent of the damage. Furthermore, different monitoring methods have potentials to evaluate specific damage and multi sensor approaches have raised the attention of the community as well [11]. As a consequence, the necessity of taking transducer on board is a must to correctly operate system monitoring. All this can be extended to aircraft systems, such as actuators, where either transducers already available or new ones can be adopted to explore the state of health of the system [12].

However, to get track with the integration of SHM systems, it is pivotal understanding both the reliability and the affordability of such a system. Indeed, the lack of reliability and cost-benefit assessment still limit the industrial deployment of on-condition maintenance systems. The reliability is strongly depending upon the specific SHM approach adopted and it has been increasingly approached during the last years in tight collaboration among experts of SHM approaches and reliability [13]. Actually, it can help deriving the minimum detectable size of a damage during lifetime, returning the target of a specific SHM system and, as such, the level of implementation within aircraft operative life. On the other hand, cost-benefit analysis is challenging in the way to estimate both the benefit (in terms of maintenance, structural design, or both) and the cost (in terms of system revenue and mass increase). Hence, it is necessary to introduce a multidisciplinary analysis which accounts both the aspect of health management and influencing parameters during design and performance assessment. This analysis is independent upon the specific SHM technique and technology adopted, as it needs the sources of cost and benefit as aseptic inputs. As a matter of fact, this will provide the roadmap for efficient integration and implementation of SHM systems, driving in an inverse manner the design of such a system to be profitable rather than effective only.

As to the design, one of the main issues of recent aircraft lies in the novel materials adopted for the design of load bearing structures, i.e., composites. Although they allow tailoring the properties of the structural component with net performance improvement, the level of anisotropy introduced is quite critical. In particular, the stacking characteristics of a laminate and the lower strength in the normal direction make the composite materials failing under dynamic loads normal to the surface. This is often caused by complex mechanics developing locally and resulting in the loss of continuity among adjacent layers (i.e., delamination) because high interlaminar shear stress arises [14,15,16]. What is more dangerous consists in the random occurrence of low velocity impacts which may produce failure and threaten the aircraft mission if not addressed appropriately. The safety critical aspect requires an opportune design, which is usually driven by the damage tolerance approach, currently highlighting the necessity to accommodate the material allowable accounting for undetectable damage presence. This begs the question about saving weight by making those damage types detectable in an efficient manner.

The safety by design philosophy adopted in the aviation field was encompassed in early 80s’ by the damage tolerance approach rather based on the use of inspection to ensure safety along with structural design concepts to protect safety while following inspection procedures [17,18,19]. However, this safety by inspection approach still fails to properly exploit material capabilities as it is strongly affected by the sensitivity of inspection procedures and cost thereof. As a consequence, maintenance tasks are costly because they require detailed inspection of hidden failures and a longer downtime. The result is an increasing direct operating cost (DOC) due to exceeding safety weight and safety critical operations. That is where SHM can benefit airliner costs from a conceptual standpoint. SHM aims at interrogating the aircraft continuously to warn the presence of any damage, which can be monitored resorting to a variety of approaches [20,21]. Postulated that SHM integration can enable the detection of damage below allowable damage limit, then the benefit relies on one hand, on a more flexible and faster maintenance schedule along with an increasing level of safety and, on the other hand, on more relaxed design.

About the first advantage, Fioriti et al. [22] realized that effective prognostics can make the aircraft more available increasing airliner profit. However, it is extremely critical the way to exploit the value of SHM implementing the diagnostic/prognostic-derived information in the health management to enable a condition-based maintenance. Generally, there is already a few discussions about integration of real-time monitoring and relative cost and benefit [23,24]. However, this is far from realistic, being based on the simultaneous use of wireless connection, onboard sensory system and computing base station located on the ground. However, overcoming technology gaps in SHM operation compliant with aircraft operation, SHM is expected to prevent Visual and Non-Destructive Inspection (V-NDI). Indeed, the detailed visual inspection causes the 80–90% of maintenance downtime, while the remaining out of service aircraft is derived by non-destructive inspection and health management [25]. All these tasks can be strongly limited ensuring the intervention of the expert operators only when the SHM system wars possible anomalies. Nonetheless, the investigation carried out is not really promising when approaching an ageing aircraft, the Boeing B737NG, where only the maintenance can benefit from SHM. Indeed, it turned out that a large number of piezoelectric transducers is required to be taken in place of a traditional C-check. In details, the SHM system allows reducing the maintenance downtime and, as such, the maintenance cost. However, the achievable benefit is much lower than the operating cost penalty generated by the sensors system weight. Hence, it turned out that a cost-effective SHM would be achievable either improving the current sensor technologies so that fewer sensors are needed or adjusting the aircraft design concept according to SHM.

On the other hand, Dienel et al. [26] had a first look at the benefit achievable implementing SHM into the design loop introducing more relaxed design constraints relying to continuous monitoring. The authors estimated a 9% weight relief achievable thanks to the SHM system based on ultrasound by adjusting the current damage tolerance criterion to satisfy smaller defect. This directly leads to reduction of structure thickness. However, the net weight saving is around 5% taking into consideration the mass of the SHM system. In addition, a detailed investigation provided by the authors showed that multidisciplinary analysis can be used to outfit a range of options according to number of sensors and achievable benefit [27]. In particular, according to the transducer density over the airframe, the SHM system can either reduce or increase DOC and the limit for this define the breakeven point for the feasibility of a specific SHM technology. In other words, the authors laid down a multidisciplinary formulation to make clear the minimum number of sensors (or weight) taken on board to satisfy affordability. However, the advantages of SHM-driven design were not accounted for. Therefore, the efforts made by the authors in assessing the performance of an SHM system and the advantages in integrating such a technology within airframe does not still return enough results to make a quantitative assessment of the benefits at aircraft level, demanding for the investigation of introducing this new design approach for the systematic quantification thereof.

To fill the knowledge gap in the available literature, it is needed to assess the maximum mass that could be added to a newly conceived aircraft, whose design is moved by the value of information provided by SHM, in order to reduce operating costs without any significant performance loss. Within this context, the aim of this work is to establish a parametric study based on multi-disciplinary analysis returning the impact of the SHM system at aircraft level encompassing a SHM-driven design.

The remaining of the paper is organized as follows. Materials and Methods describes the methodology adopted to calculate costs and benefit of SHM. Instead, Results section show the several assumptions made along the quantitative outcomes of the investigation in integrating SHM technology at aircraft level. Finally, a brief discussion about concluding and future remarks closes the paper.

## 2. Materials and Methods

In this section the methodology and the approaches used to estimate the potential benefits of introducing a Structural Health Monitoring system will be illustrated.

The objective of this research work is to estimate the benefits in terms of aircraft DOC given by the introduction of a SHM system that could potentially lead to a reduction of the safety margin usually imposed by the regulations when composite materials are envisaged for aircraft structures. A brief overview of the applied multidisciplinary workflow and is provided together with and insight of the methodologies used to estimate aircraft Direct operating Costs and the specific analysis about how a safety margin reduction impact on structural sizing.

### 2.1. Multidisciplinary Workflow and Performance Estimation

A reliable evaluation of the potential benefits brought by the SHM system need to be assessed at aircraft level through a multidisciplinary analysis in where all the required branches are simultaneously involved. Starting from, the definition of a reference aircraft the multidisciplinary approach will address the aircraft weight estimation (including the impact the SHM system have on the aircraft structural mass breakdown), the weight and balance, the aerodynamic dataset, aircraft performance and finally the direct operating costs.

The mass estimation of weights and balance of each aircraft component, onboard systems are performed using the formulations and approaches suggested by Torenbeek in [28]. The impact the application of composite materials has on wing and fuselage structure is assessed through a specific analysis at material level then applied to the whole structural mass estimate at component level. Same approach is used to evaluate the impact the safety margin has on the structural sizing of the key structural elements of both the wing and the fuselage components.

The aircraft aerodynamic database is built by integrating simple and reliable semi-empirical formulation like those proposed by Roskam [29], Raymer [30] and Sforza [31].

**Figure 1:**
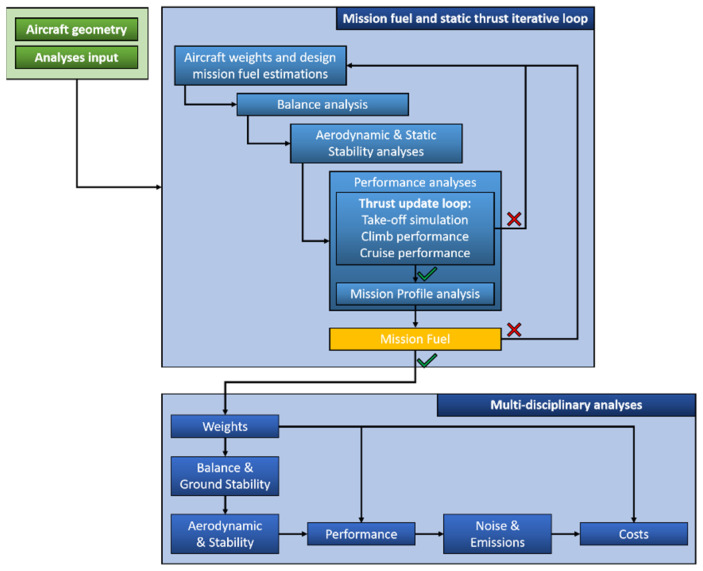
Multidisciplinary workflow including thrust update inner loop, reprinted with permission from [32].

The workflow of this multidisciplinary analysis is sketched in the chart of Figure 1. The key part of that workflow is the converger loop about the aircraft weights estimation.

According to the design mission (or the assigned mission to be analyzed), there is the need to get the convergence on the required fuel to fulfill the mission profile. This loop gathers several aspects: a first educated guest of the aircraft weights breakdown, the balance analysis, the aerodynamic and stability of the aircraft, the flight performance to calculate the required fuel and the required thrust to match the assigned Top Level Aircraft Requirements (TLARs).

Once the converge is reached, the updated weights, balance, aerodynamics are used to feed the final performance estimation reaching the converged outputs (including noise and emission). This is accomplished through JPAD (Java toolchain of Programs for Aircraft Design). JPAD is a software developed at the Industrial Engineering Department of the University of Naples Federico II (Napoli, Italy) [32,33,34,35].

In this research work, the inner loop about the engine thrust update will be not activated since to highlight the net effects at aircraft level of the introduction of SHM technology. This enables the possibility to estimate the net effects on aircraft performance due to the additional mass introduced by the application of permanently attached monitoring sensors and the potential weight savings due to a reduced safety margin applied to the key wing ad fuselage structural elements.

### 2.2. Insight of Direct Operating Costs Estimation

**Figure 2:**
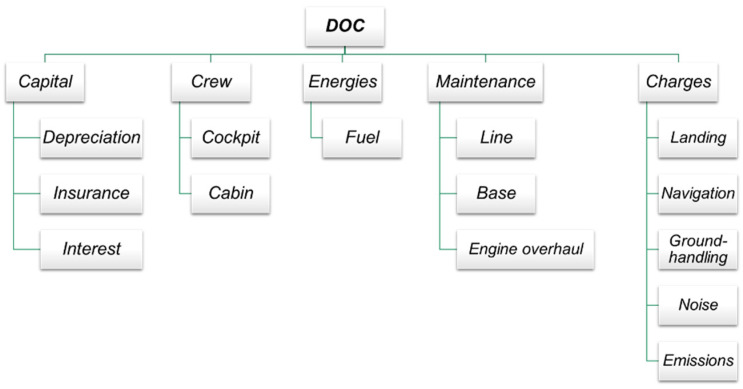
DOC breakdown.

A cost estimation plays a fundamental role since the very beginning of a new aircraft design process, in order to assess the market competitiveness. A complete cost analysis shall evaluate the amount of resources involved during the whole life cycle of the product under study, considering the cost of developing, producing, operating, and disposing it. Operating costs represent the highest costs of the whole life cycle and therefore an estimation already at a conceptual-preliminary phase of the aircraft project is extremely important. Each airline develops its own methods for estimating operating expenses related to their operation, flight patterns, aircraft fleet and accounting procedures. Since these methods vary widely between different operators it is necessary, during design process, to use a standardised method for cost analysis, especially in the early stages of the design [36]. The methodologies for cost estimations have been already widely descripted by the authors in [27]. The scheme is in Figure 2, while Table 1 resumes the key cost relationships for DOC calculation. To assess the economic impact of the innovative configuration, a complete analysis of the Direct Operating Cost (DOC) has been performed. Moreover, the outcomes of direct operating costs investigations have been exploited to compare different configurations coming from different assumptions on technology of SHM system.

In particular, the effects of SHM on cash DOC, which is defined as the DOC less the Capital costs, has been stressed. The reason lies on the fact that airliners could prefer to lease an aircraft instead of to buy it. Moreover, the capital costs are usually the main part of direct operating costs, therefore the impact of maintenance costs reduction due to SHM couldn’t emerge from DOC perspective. In the following, a schematic resume of the equation for the DOC calculation.

### 2.3. Insight of SHM Impact on Potential Safety Margin Reduction

The impact of SHM at aircraft level is manly twofold. First of all, it enables the avoidance of any type of unscheduled maintenance, the limitation of scheduled maintenance to a few critical cases and the adoption of predictive philosophy as standard procedure during lifetime. This process, based on a sort of on-condition approaches, can strongly reduce the direct operating cost of the aircraft due to maintenance tasks currently prescribed by damage tolerance design. However, the disruptive implementation of SHM relies on the modification of the design starting from the value of the information given by the SHM. The impact that this latter can produced implementing a new paradigm of structural design can be outlook summarizing what is emerging analyzing a real certification stage of an aeronautical composite structure. As prescribed by certification handbooks [17,18,19], visible and barely visible damage need unaffecting the safety of the aircraft in between three following inspection checks whenever they occur during lifetime.

**Figure 3:**
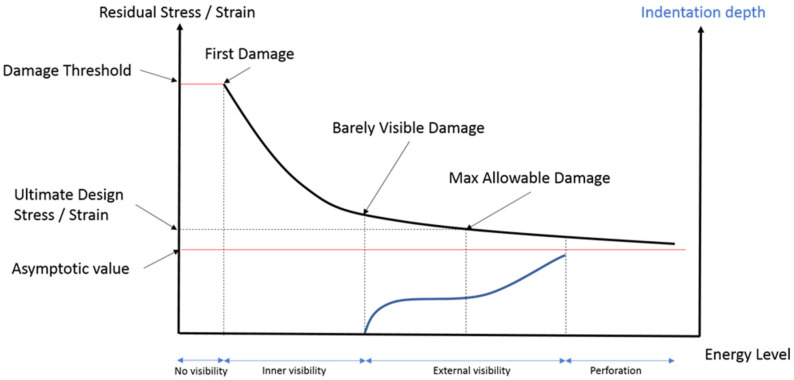
Schematic representation of residual strain versus energy level and derived design constraints.

To detail how the safety design is influenced by unforeseen events, it is worth looking at the typical trend of residual stress/strain versus impact energy level, which is qualitatively schematized in Figure 3. When the energy of the impact is very low, no visible damage is present as the phenomenon does not ensure the onset of any hidden failure. Increasing the energy, a first damage appears, and the residual strain/stress of the material start to decrease deeply despite the damage is still barely visible. The failure can be characterized by only through thickness damage in form of delamination (inner visibility) or hidden flaw is present along with slightly visible indentation (external visibility). This latter is not always appreciable by visual inspection and define the maximum allowable damage according to the ultimate design stress/strain associated with residual stress/strain. A further increase of energy, the damage becomes wider and more visible, with no hidden peculiarities. However, most of the residual strain/stress is already lost and a knockdown factor due to the presence of barely visible damage needs to be included in the design regardless any other safety factor.

**Figure 4:**
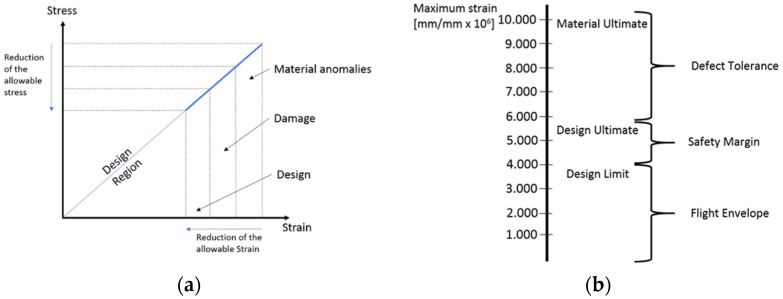
Schematic of scattering factors constraining the composite design (**a**) and Typical allowable design for CFRP (**b**).

In addition to damage, other aspects should be accounted in the design of the composite components affecting the allowable, as reported in Figure 4a, whose scheme shows the design region enabled via increasing reduction of allowable stress/strain due to several material anomalies (voids, humidity, etc.), barely visible damage and safety margin. Actually, composite materials are designed considering limit allowable, which is appreciably lower than material ultimate due to the tolerance introduced (Figure 4b according to Boeing Design Manual and Military Handbook [17,18,19,44]). Consequently, the design limit allowable σ_d.l.a._ can be calculated for first approximation introducing a scatter to the ultimate material allowable σ_m.u.a_ as follows:

$$\sigma_{d.l.a}\;=\;0.5\;\times \;\sigma_{m.u.a.}$$

What can be disruptive is that SHM can reduce the scatter, currently close to 2 and adjust the design to achieve a lighter structure. To achieve this breakthrough, it is crucial implementing a reliable SHM system able to continuously monitor the airframe and aircraft systems in every location resorting to distributed sensor networks. Likewise, it is important to address the affordability of the SHM integration and under which conditions having it on board becomes profitable. Without altering the generality of the further discussion, the analysis requires of combining possible benefits and costs due to an SHM system, no matter the specific approach adopted. To perform such a holistic analysis, the system must be considered aseptically and including the main benefit expected and the possible weight and cost resulting. In this way, the findings will be pivotal to design an effective and profitable SHM system based on any specific technique.

## 3. Results

The main objective of this work is the estimation of the possible impact of the SHM system on the aircraft design, in particular on the airframe characteristics. In fact, the authors would like to explore the possible gain in DOC and aircraft performance due to weight reduction coming from a new assumption on safety margin which could be possible if SHM is installed on board and can reveal the presence of barely visible damage currently affecting design inducing knockdown factors. The key limitation in maximizing the potential benefits of such a technology is represented by the regulation imposed in sizing aircraft composite structures. In fact, a safety margin of 2.0 is roughly applied to estimate the ultimate loading that composite structures must withstand. This is because there is not any inspection which can ensure the detectability of barely visible damage under a certain visibility range. However, a structural health monitoring system should be a viable way for a real time check for the health status of a composite structure and reduce the minimum detectable size of defect during lifetime. Thus, SHM implementation can help into reducing the stringent safety margin imposed by aviation regulation for a still safe design of composite structures. By assuming a safety margin reduction from 2.0 to 1.75, this paper wants to assess the potential fuel savings and direct operating costs through a multidisciplinary analysis on a A220-like aircraft.

In this respect, the authors have already been measured the possible reduction of DOC by implementing SHM, through a multidisciplinary analysis framework which compared the increment of aircraft operating empty weight with the possible benefits in terms of direct operating costs due to reduction of the maintenance costs [27]. A parametric study was performed assuming the sensor density as input variable of the SHM system. This procedure allows to identify the breakeven point between the aircraft MTOW (increased by sensors’ mass) and the variation in aircraft DOC (mainly modified by the maintenance costs and sensor integration). The break-even point for the SHM technology was estimated about 30 sensors per square meters: this means that in case of the SHM integration on A220-like aircraft, there will be a higher OEW (+7%) but the DOC should not. Starting from this outcome, this research assumes a different safety factor for the structural design, equal to 1.75 while for composite it should be equal to 2 (as in the previous paper was done). This assumption should imply a lower OEW with a consequent gain in terms of DOC even with 30 sensors per square meters.

In a big picture, the introduction of SHM technology should involve in complete re-design activity to ensure the same performance as field lengths, time to climb, block fuel, block time, emissions, and so on. To get the most out of such a technology, a multidisciplinary optimization should be accomplished at aircraft level to define the optimum aircraft solution compliant with a specific set of top-level requirements aiming at the minimum DOC or at the minimum environmental impact. However, one of the main objectives of this research, was to make as much knowledge as possible for the future activities about optimization at aircraft design level.

Before to show the results in terms of weight, performance, and DOC, it should be useful to report the main characteristics of the jet aircraft selected as reference platform like the Airbus A220-300. The reference aircraft is modeled through JPAD software assuming the set of TLARs of the Airbus A220-300. The main aircraft characteristics are summarized in Table 2.

The aircraft model was already validated in the previous research [27]. For the sake of clarity, the comparison is reported also in the following Table 3. In the same research, the cost model for all direct operating items was described in detail. Herein, it will be reported just the main important economic assumptions following the scheme in [27].

Table 4 and Table 5 report respectively the economic assumptions, weights and performance data used for the comparison.

Data in Table 5 refer to the reference aircraft without SHM sensors, to be exact. In this paper, it was necessary to define a new reference configuration with the maximum number of sensors allowed, let’s say the higher sensors density that doesn’t involve in a negative DOC difference with respect to aircraft configuration without SHM system [27]. Moreover, another important aspect to underline is the lack of knowledge about the best value of sensors density (it was unknown a priori), since it highly depends on the technological level.

Then, in a first step of the assessment it was investigated the impact of the reduction of the structural safety margin on the airframe weight and consequently on aircraft performance. In this respect, to promote the introduction of SHM at aircraft system level by reducing the structural safety margin, a change of the regulations should be necessary. In fact, the aim of the SHM could be a reduction of airframe weight, which not only balance the mass added (i.e., the sensors) but could also involve in a possible further gain in terms of weight and consequently in aircraft performance (including DOC). As already shown by authors in [27], the impact of SHM sensors does not apply linearly to aircraft OEW or WTO: 1 kg of SHM do not correspond 1 kg of additional mass of OEW or WTO. This latter highlights how the aircraft mass snowball effect impacts on the estimation of the DOC net benefits due to the SHM.

Table 6 shows how every aircraft component mass change by assuming the two different SHM sensors densities related to the old and the new reference configuration. In order to make a realistic assumption of the mass added on board according to the density of sensors integrated, an assumption is made which ensures to encompass most of the system relying on distributed sensors networks and it is based on the use of small and light transducers, such as piezoelectric sensors. To manage them, it is necessary to wire the whole aircraft (including cables and connectors) and integrate power electronics to control the system and deal with data acquisition. In addition to cost of the equipment and its installation, it is worth accounting for the mass moved on board accordingly. This has been assumed considering the typical configuration described above and distributing the whole mass to the number of transducers. The mass per sensor (around 80 g) returns a penalty to be extended at aircraft level according to [27], which returns Table 6. Instead, Table 7 summarizes the aircraft performance and DOC against the density of sensors adopted.

It was assumed that the SHM technology could lead to a reduction of maintenance cost of the 50% about line maintenance and 50% of the base maintenance costs, while SHM system does not impact on the engine overhaul costs. Again, to achieve the assumed reduction of the maintenance cost, the number of required sensors per square meter is unknown. This value relies on the specific methodology adopted to detect damage and apply health management. That is to say, the mass of the new system that is integrated within the aircraft, has been defined in a such way that does not exceed the mass increment at the break-even point [27], preventing significant performance loss. Table 8 shows the link between density and estimated costs per components. It is interesting to note that just the fuselage induces more than 50% of the added weight.

**Figure 5:**
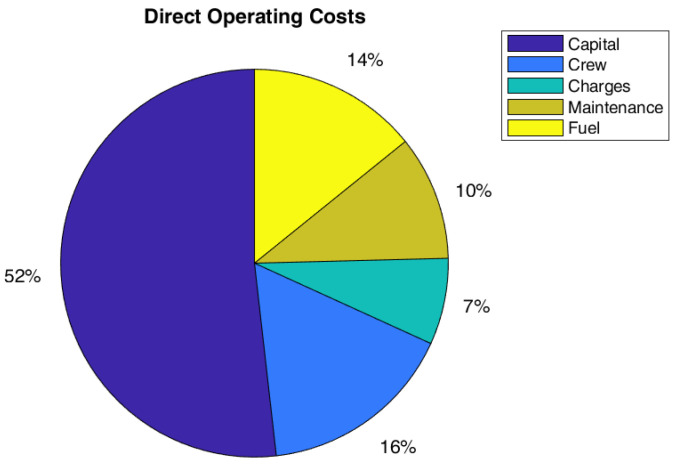
Pie chart of DOC for Configuration A (SM = 1.75, with SHM).

Starting from the new reference configuration with SHM, the airframe was re-designed considering a value of safety margin SM equal to 1.75, as stated previously. Table 9 compares the new weight breakdown for a named Conf. B with the previous aircraft configurations (i.e., the reference and Conf. A). The structural weight reduction brought by a reduced SM for the Config. B becomes an enabler for reducing costs and emissions without impacting the overall performance (in particular the take-off and landing distances), as reported in Table 9 and Figure 5.

**Figure 6:**
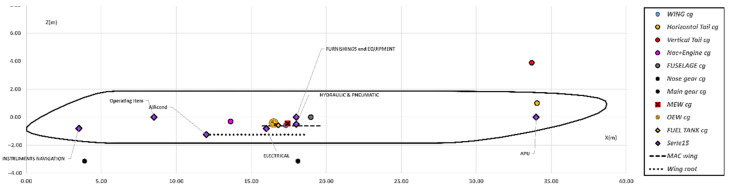
Centre of gravity position of each aircraft component and system.

By looking at Table 9 and Table 10, the configuration named Conf. B exhibits a slightly increase in the maximum take-off weight with respect to the reference aircraft, whereas the take-off field length is significantly reduced (−13% with respect to the reference one). The impact the safety factor has on the structural weight reduction is a different effect for each aircraft component. Since, the analysis framework is a multidisciplinary environment, the differential impact on aircraft components mass is reflecting also on the aircraft weight and balance analysis. The positions of each of aircraft component and system center of gravity is shown in Figure 6, they have been estimated according to Torenbeek’s suggestions [28]. These center of gravity positionings are the same for all the investigated aircraft configurations, whereas the impact each one has on the estimate of the Operative Empty Weight (OEW) center of gravity position is different since components and systems weight change accordingly to the considered configuration. The position of the center of gravity in the OEW condition, is the starting point from which the boarding diagram is built. The boarding diagram allows the calculation of the range of the center of gravity travel according to cabin aisle (3-2 for the investigated A220-like aircraft), the average passenger weight (103.5 kg per pax is here assumed) and the fuel needed to fulfill the mission profile.

**Figure 7:**
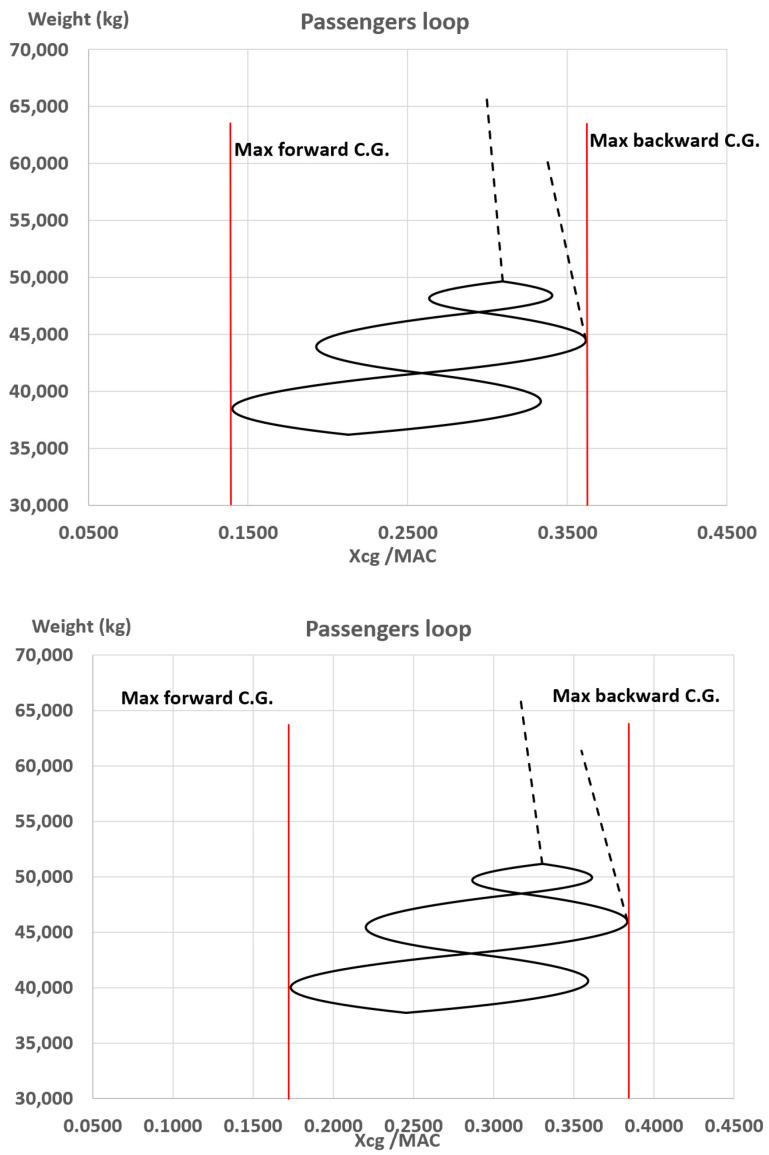
Centre of gravity excursion range: Ref. configuration (**up**) vs. Conf. B (**low**).

The comparison between the center of gravity excursion range of the reference aircraft and the configuration B is shown in the chart of Figure 7.

From the chart of Figure 7 it can be appreciated how the excursion range is ~21% of the wing mean aerodynamic chord for both the configuration, but in the case of Conf. B the max backward center of gravity position is at ~38%MAC whereas the in the Ref. aircraft this is placed at ~36%MAC affecting the aircraft longitudinal Static Stability Margin (SSM). Both configurations are naturally stable with a SSM higher than 5% (in terms of wing mean aerodynamic chord) but Conf. B shows a SSM lower than 2% with respect to the reference one. It must be here remarked that aircraft components sizing, and relative positioning is not being changed in this investigation. Thus, a reduction of SSM results in a better controllability of the aircraft that, in turn, means a lower downforce on the horizontal tail to trim the aircraft. The latter has almost a negligible effect concerning the cruise conditions, where the required tail downforce is low, but in take-off conditions (low speeds and deployed high lift devices) a lower SSM can bring to a relevant reduction of the tail downforce needed to trim the aircraft at high attitude angles leading to higher maximum lift coefficient that positively affect the take-off field distance.

**Figure 8:**
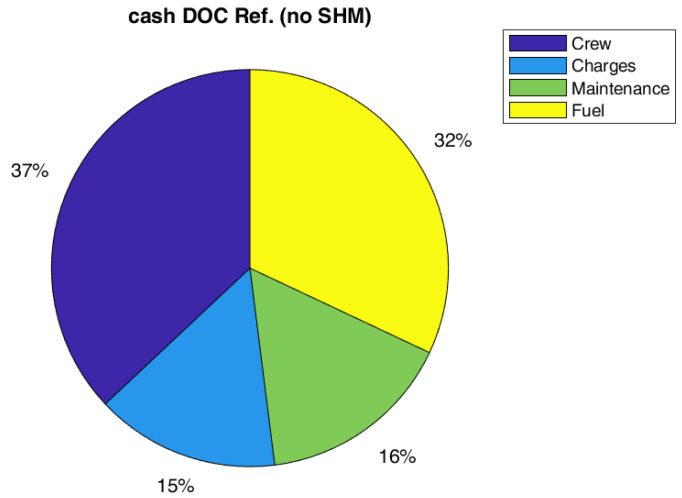
Pie chart of cash DOC for the Ref. configuration without SHM (SM = 2.0).

**Figure 9:**
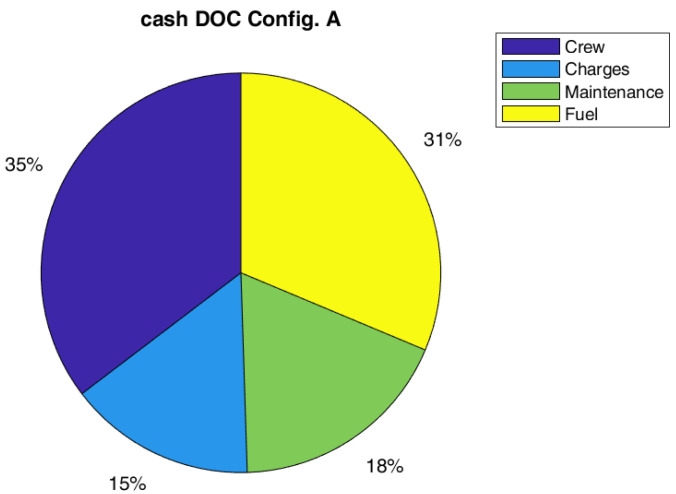
Pie chart of cash DOC for Config. A (SM = 2.0, with SHM).

**Figure 10:**
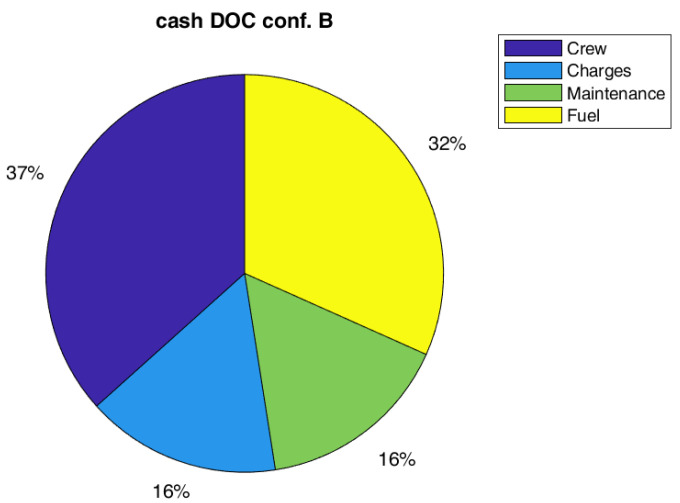
Pie chart of cash DOC for Config. B (SM = 1.75, with SHM).

Regarding costs, the effect on global DOC is not so relevant, a reduction of 1% on the global value has been estimated. However, the global value of DOC could be not the best metric to measure the impact on direct costs. In fact, the capital costs (depreciation, interest, and insurance costs) are the main contributions to DOC, and they are not highly affected by SHM and therefore, it could mitigate the effect of maintenance cost reduction due to SHM (even if there should be a small positive effect of the weight on capital costs). In this respect, it could be interesting to also report the effect of SHM introduction on cash DOC, which is the DOC fewer capital costs. Indeed, it could be really interesting such kind of analysis since several airlines prefer to lease instead of to buy an aircraft. As in Table 11, the possible gain of the SHM could be estimated equal to 11% for both configurations, A and B. The relative importance of cost items considering the cash DOC is practically the same for all configurations, as shown in Figure 8, Figure 9 and Figure 10.

In order to obtain a greater effect in terms of direct operating costs, it has been investigated also the configuration with a lower value of sensors density, equal to 10 sensors per square meters, and designed with a lower value of safety margin, equal to 1.75, named configuration C. Again, the investigation based on this assumption could be considered as trend analysis, since the best density was unknow as already stated above and it strongly depends on the technology level of the SHM system. As already done for other configurations, Table 12 describes the SHM system costs and weight, Table 13 compares the impact of the sensors on aircraft weight components, while Table 14 and Table 15 assesses the impact of SHM system in terms of performance and operating costs for configuration C.

In this case, the gain in DOC is higher with respect to previous configurations, probably due to OEW like configuration without SHM, which entails in similar capital costs of reference configuration, but with the advantages on maintenance costs related to SHM of Configurations A and B. Furthermore, also performance are in line with the reference configuration without SHM system and with safety margin equal to 2.0.

**Figure 11:**
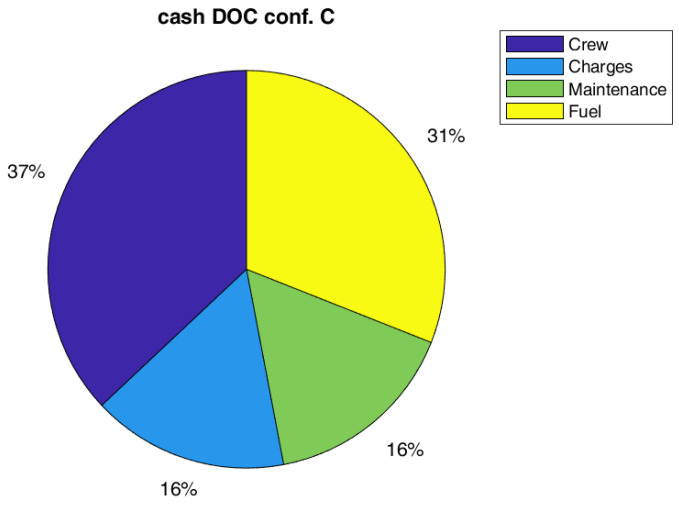
Pie chart of cash DOC for Configuration C (SM = 1.75, with SHM—10 sensors per m^2^).

In a big picture, the combined assumptions on reduced density sensors and safety margin, allow to obtain an aircraft like reference aircraft (without SHM). Therefore, these value of sensors density and the safety margin could be considered as a possible target for SHM system design. Even analyzing the cost breakdown in Table 15, Configuration C seems to be the most promising one, with a reduction of all cost items. In terms of relative importance of the cost items, C is in line with the other configurations analyzed (Figure 11). As a final remark, it is worth noting that the assumption made on the SHM system does not alter the generality of the discussion. Considering the SHM system mass as entry input, the result will be quantitatively the same. Actually, the type of sensors and installation may slightly vary the mass per sensor and move the profitable condition to a different sensor density, without changing the results qualitatively and keeping the same most promising configuration.

## 4. Conclusions

The introduction of SHM technology enables to introduce an on-demand screening of the current airframe health increasing flight safety during the whole aircraft lifetime. This technology could represent a viable way to allow for a reduction of the safety margin imposed by certification authorities to design safe composite structures. Starting from a reference platform, represented by an A220-like jet civil aircraft, a reduction of safety knockdown factor in designing aircraft structures from 2.0 to 1.75 has been foreseen. Through a multidisciplinary framework, the potential benefits due to the structural weight reduction has been assessed in terms of performance, direct operating costs and emissions. Three configurations have been investigated and compared to the reference aircraft: (i) an A220-like aircraft embedding a SHM system with a sensors density equal to 30 sensors per square meter for which no safety margin relief has been considered; (ii) the same configuration for which a reduction of safety margin from 2.0 to 1.75 has been envisaged and (iii) same configuration of the second case with a lower sensors density (i.e., 10 sensors per square meter).

Results indicate that the application of a SHM to modify the maintenance procedure can lead to a 3% DOC reduction (compared to the reference civil jet aircraft). However, the overall structural weight is increased due to the presence of the permanently attached sensors, affecting both aircraft flight performance, with the most detrimental impact on the take-off field length (+8%) and emissions (+3% of block fuel). However, implementing and SHM-driven design of the airframe by reducing the safety margin to 1.75, it is possible to balance the weight increase due to sensors installation with a reduction of the structural weight. The latter leads to an almost equivalent aircraft in terms of maximum take-off weight, achieving a DOC reduction of about −3%, a block fuel saving of about −2% and no detrimental effects on the aircraft performance. A further investigation concerning the number of sensors per square meter has been performed. This investigation would be representative of the technology level of the SHM system (allowing for a reduction\elimination of some maintenance tasks) which affects procedures and costs. By reducing the sensors density from 30 to 10 per square meter, it is possible to restrain the weight increment due to sensors installation and, thanks to the relief of the safety margin, an aircraft equivalent to the reference one both in terms of wight and performance has been achieved. This solution yield to a −2% of block fuel with a potential DOC reduction of about 5%.

Although SHM still needs to demonstrate to be a mature enough technology to allow for a safety margin reduction in sizing aircraft composite structures, its implementation can provide radically new perspective in designing brand new aircraft. Indeed, by granting a relief on the structures safety margin it would be possible to maximize the impact of SHM technology towards DOC and fuel saving by means of a multidisciplinary design and optimization process in which the introduction of SHM can be envisaged from the very beginning of aircraft design phase and not only in a retrofitting fashion approach starting form a reference aircraft.

## Figures and Tables

**Table 1:** Summary of Cost Estimation Relationships for DOC calculation.

ITEM	CERs	Notes
Capital costs [37]	${\mathrm{DOC}}_{\mathrm{dep}}\;=\;\frac{\mathrm{TI}}{\mathrm{DP}}\times \left(1-\mathrm{RV}\right)$	Depreciation Period (years) Total Investment (sum of aircraft price and the spare costs) Residual Value DOC of depreciation per year
Capital costs [37]	${\mathrm{DOC}}_{\mathrm{int}}\;=\;\mathrm{TI}\;\cdot \;r_{i}$	Total Investment Annual rate DOC of interest per year
Capital costs [37]	${\mathrm{DOC}}_{\mathrm{ins}}\;=\;\mathrm{ADP}\;\cdot \;r_{a}$	Aircraft Delivery Price (sum of the airframe and engines prices) Annual rate DOC of insurance per year
Fuel costs	${\mathrm{DOC}}_{\mathrm{fuel}}\;=\;P\;\cdot \;m_{f}$	Fuel Price Fuel Mass DOC fuel
Charges: landing, navigation, ground-handling, noise, and emissions costs [38,39]	$\begin{array}{cc} {\mathrm{DOC}}_{\mathrm{charges}}\;=\; & K_{\mathrm{eco}}\;\cdot \;{\mathrm{DOC}}_{\mathrm{ldg}}\;+\;{\mathrm{DOC}}_{\mathrm{nav}}\;+\;\\ & {\mathrm{DOC}}_{\mathrm{grd}}\;+\;{\mathrm{DOC}}_{\mathrm{noise}}\;+\;\\ & {\mathrm{DOC}}_{\mathrm{emissions}} \end{array}$	Keco is set either to 0.635 or to 1.0, whether the noise and emission charges are considered or not [35]
Charges: landing [39]	${\mathrm{DOC}}_{\mathrm{ldg}}\;=\;\mathrm{MTOW}\;\cdot \;K_{\mathrm{ldg}}$	Unit rate (US $/t) equal to 7.8 for short-medium range and to 6 for long range Maximum Take-Off Weight DOC landing
Charges: navigation [39]	${\mathrm{DOC}}_{\mathrm{nav}}\;=\;R\;\cdot \;K_{\mathrm{nav}}\;\cdot \;\sqrt{\frac{\mathrm{MTOW}}{50}}$	Unit rate (US $/km × √t) equal to 0.5 for short-medium range and to 0.17 for long range Distance factor Range (km) DOC related to en-route navigation charge
Charges: ground-handling [39]	${\mathrm{DOC}}_{\mathrm{grd}}\;=\;\mathrm{PL}\;\cdot \;k_{\mathrm{grd}}$	Unit rate (US $/t) equal to 100 for short-medium range and 103 for long range Payload DOC related to ground-handling charges
Charges: noise [40,41]	${\mathrm{DOC}}_{\mathrm{noise}}\;=\;C_{\mathrm{noise}}\;\times \;\left(10^{\Delta_{a}}-10^{\Delta_{d}}\right)\;\mathrm{where}\;\Delta_{a}\;=\;\frac{L_{\mathrm{approach}}\;-\;T_{a}}{10}\;\Delta_{d}\;=\;\frac{\frac{L_{\mathrm{flyover}}\;+\;L_{\mathrm{lateral}}}{2}\;-\;T_{d}}{10}$	C_noise_: Unit noise rate ($) L_approach_: Certified noise level—approach measure point (EPNdB) L_flyover_: Certified noise level—approach measure point (EPNdB) L_lateral_: Certified noise level—lateral measure point (EPNdB) T_d_: departure airport threshold noise (EPNdB) T_a_: arrival airport threshold noise (EPNdB) DOC_noise_: DOC related to noise emissions
Charges: emissions [42]	${\mathrm{DOC}}_{\mathrm{NOx}}\;=\;C_{\mathrm{NOx}}\;m_{\mathrm{NOx},\;\mathrm{LTO}}\;a\;\;\mathrm{where}:\;\left\{\begin{array}{c} a\;=\;1\mathrm{if}\;\frac{m_{{\mathrm{NO}}_{X},\mathrm{LTO}}}{T}\;\leq \;19.6\\ a\;=\;\frac{\frac{m_{{\mathrm{NO}}_{X},\mathrm{LTO}}}{T}}{19.6}\mathrm{with}a_{\max}\;=\;4 \end{array}\right.$	Unit rate (US$) for Nox mass of NOx emitted during LTO kg DOC related to NOx emissions
Crew costs	${\mathrm{DOC}}_{\mathrm{cockpit}\;\mathrm{crew}}\;=\;{\mathrm{LR}}_{\mathrm{cockpit}}\;\cdot \;n_{\mathrm{cm}}\;{\mathrm{DOC}}_{\mathrm{cabin}\;\mathrm{crew}}\;=\;{\mathrm{LR}}_{\mathrm{cabin}}\;\cdot \;n_{\mathrm{cm}}$	Labour Rate Number of crew member
Maintenance costs [43]	$\begin{array}{cc} {\mathrm{DOC}}_{\mathrm{mintenance}} & =\;\mathrm{Line}\;\mathrm{maintenance}\;\mathrm{costs}\\ & +\mathrm{Base}\;\mathrm{maintenance}\;\mathrm{costs}\\ & +\mathrm{Engine}\;\mathrm{overhaul}\;\mathrm{costs}\\ & +\mathrm{Burden}\;\mathrm{costs} \end{array}\;\begin{array}{cc} {\mathrm{DOC}}_{\mathrm{Line}\;\mathrm{maint}} & =\;59.359\;-\;0.0154\;\times \;\mathrm{fleet}\;\mathrm{size}\\ & +\;9.9939\;\times \;U\;+\;28.325\;\cdot \;\left(\frac{\mathrm{FH}}{\mathrm{FC}}\right)\\ & -\;1.4008\;\times \;{\mathrm{age}}_{\mathrm{av}} \end{array}\;\begin{array}{cc} {\mathrm{DOC}}_{\mathrm{Base}\;\mathrm{maint}} & =\;44.519\;-\;0.0116\;\times \;\mathrm{fleet}\;\mathrm{size}\\ & +\;7.4954\;\times \;U\;+\;21.244\;\times \;\left(\frac{\mathrm{FH}}{\mathrm{FC}}\right)\\ & -\;1.0506\;\times \;{\mathrm{age}}_{\mathrm{av}} \end{array}\;\begin{array}{cc} {\mathrm{DOC}}_{\mathrm{Eng}\;\mathrm{overhaul}} & =\;135.16\;-\;19.754\\ & \times \;U\;-\;0.0189\;\times \;{\mathrm{age}}_{\mathrm{type}\;\mathrm{AC}}\\ & +\;11.72\;\times \;N_{e}\;+\;0.0055\;\times \;T \end{array}\;{\mathrm{DOC}}_{\mathrm{Burden}}\;=\;\frac{{\mathrm{DOC}}_{\mathrm{Line}\;\mathrm{maint}}\;+\;{\mathrm{DOC}}_{\mathrm{Base}\;\mathrm{maint}}\;+\;{\mathrm{DOC}}_{\mathrm{Eng}\;\mathrm{overhaul}}}{0.6}$	U: Utilization (h/day) FH: Flight hour FC: Flight cycle age_av_: Aircraft average age (years) age_typeAC_: Age of type of aircraft (months) Ne: Number of engines T: Thrust (lbf) DOC _Line maint_: DOC related to Line maintenance DOC _Base maint_: DOC related to Base maintenance DOC _Eng overhaul_: DOC related to Engine overhaul DOC _Burden_: DOC related to Burden DOC _maintenance_: DOC related to total direct maintenance

**Table 2:** Main data concerning the A220-300, reprinted from [27].

TLAR	
Accommodation (Typical-Full Economy)	135
Design range (typical)	3100 NM
Take-Off Field Length (Max Take-Off Weight, ISA conditions, Sea Level)	1890 m
Landing Field Length (Max Take-Off Weight, ISA conditions, Sea Level)	1509 m
Cruise Mach number (typical)	0.78–0.80
Cruise altitude (typical)	37,000 ft
Max cruise Mach number at 37,000 ft	0.82
Max operating altitude	41,000 ft
Alternate cruise range (assumed by authors)	200 NM
Alternate cruise altitude (assumed by authors)	20,000 ft
Holding duration (assumed by authors)	30 min
Holding altitude (assumed by authors)	1500 ft/min
Residual fuel reserve (assumed by authors)	5%
**Geometrical and Operational Data**	
Wing area	112.3 m^2^
Wingspan	35.1 m
Wing aspect ratio	10.97
Fuselage length	38.71 m
Fuselage diameter	3.7 m
Single engine static thrust	24,400 lb
Engine by-pass ratio	12:1
Max Take-Off Weight	67,585 kg
Max Landing Weight	58,740 kg
Max Zero-Fuel Weight	55,792 kg
Operating Empty Weight	37,081 kg
Max Payload	18,711 kg
Max Fuel Mass	17,726 kg
BADA averaged climb speed (CAS)	271 kt
BADA averaged rate of climb	1642 ft/min
BADA maximum rate of climb	2862 ft/min
BADA averaged descent speed (CAS)	218 kt
BADA averaged rate of descent	2186 ft/min
BADA maximum rate of descent	3700 ft/min

**Table 3:** Comparison between JPAD output and A220-330 data in Table 2.

Parameters	JPAD	A220-300	Difference (%)
Max Take-Off Weight (kg)	66,956	67,585	−0.93%
Max Landing Weight (kg)	56,875	58,740	−3.18%
Max fuel Mass (kg)	17,553	17,726	−0.98%
Max Zero-Fuel Weight (kg)	53,951	55,792	−3.30%
Operating Empty Weight (kg)	36,916	37,081	−0.45%
Take-Off Field Length (m)	1837	1890	−2.78%
Landing Field Length (m)	1509	1509	0.00%

**Table 4:** Economic assumptions.

	Value	Units	Notes
Life span	16	years	
Residual value	10%		Value of the A/C at the end of operative life
No. seats	135		
Aircraft price	101.8	US$ million	It is higher than A220, to consider the impact of SHM in terms of retrofitting costs, as explained in [45]
Engine price (each)	12	US$ million	
Spares	14.9	US$ million	the cost of aircraft spare parts is assumed as the 10% of the aircraft cost, while the engine spare part as the 30% of the engine cost
Interest	5.4%	per year	
Insurance	0.5%	per year	
No. of flights	558		
Utilization	3750	h/year	Number of revenue hours per year
Block Time	6.72	h	Block Time (BT) is the total time spent from starting engines to engines off
Block Fuel (mission)	14,402	kg	
Age of type of aircraft	24	months	Age of the A/C model
Average age	1	years	Age of the A/C
Fleet size	30		
Fuel Price	1.4	US$/gal	

**Table 5:** Data for DOC estimation: reference mission data and weights.

PERFORMANCE	
16	
Years	
Range	3100 NM
Mach cruise	~0.80
SFC (Specific Fuel Consumption at cruise)	0.532 lb/(lb × h)
T_0_ (thrust)	24,400 lb
**WEIGHTS**	
MTOW	66,956 lb
OEW	36,916 lb
PAYLOAD	14,648 lb
FUEL (mission)	15,393 lb

**Table 6:** Weight estimation for different components.

	Density (nr./m^2^)	Sensor’s Weight (kg)	OEW (kg)	MTOW (kg)	Fuselage (kg)	Wing (kg)	H-Tail (kg)	V-Tail (kg)
**Ref.**	0	0	36,916	66,956	7101	6880	812	653
**Conf. A**	30	1759	39,333 (+7%)	69,717 (+4%)	8059 (+13%)	7647 (+11%)	949 (+17%)	766 (+17%)

**Table 7:** Aircraft performance and DOC at different sensors density for the design mission.

	Density (nr./m^2^)	DOC (US $/h)	TO Field Length (m)	Time to Climb (min)	M Cruise	Landing Distance (m)	Block Fuel (kg)	Block Time (min)
**Ref**	0	6518	1837	17.38	0.80	1509	13,706	401
**Conf. A**	30	6350 (−3%)	1983 (+8%)	1521 (+1%)	14,073 (+3%)	400 (−0.2%)	6350 (−3%)	1983 (+8%)

**Table 8:** SHM system characteristics.

Component	Density (nr./m^2^)	Estimated Costs (€)	Weight (kg)
Fuselage	30	2,685,698	958
Wing	30	1,549,251	552
Horizontal tail	30	384,903	137
Vertical tail	30	314,779	112
ToT.		4,934,631	1759

**Table 9:** Weight comparison for a Conf. B (SM = 1.75 instead of 2.0, thanks to SHM), with respect to Ref. configuration (see [1]) and the new reference, herein, named Conf. A (configuration with SHM, but with SM = 2).

	Density (nr./m^2^)	Safety Margin	OEW (kg)	MTOW (kg)	Fuselage (kg)	Wing (kg)	H-Tail (kg)	V-Tail (kg)
**Ref.**	0	2.0	36,189	66,953	7101	6454	812	653
**Conf. A**	30	2.0	39,333 (+7%)	69,717 (+4%)	8059 (+13%)	7647 (+11%)	949 (+17%)	766 (+17%)
**Conf. B**	30	1.75	37,760 (+3%)	67,961 (+1%)	7733 (8%)	7110 (+4%)	906 (+12%)	731 (+12%)

**Table 10:** Performance comparison the Conf. B (SM = 1.75 instead of 2.0, thanks to SHM), with respect to Ref. configuration (see [1]) and the new reference, herein, named Conf. A (configuration with SHM, but with SM = 2).

	Density (nr./m^2^)	Safety Margin	DOC (US $/h)	TO Field Length (m)	Landing Distance (m)	Block Fuel (kg)	Block Time (min)
**Ref.**	0	2.0	6518	1837	1509	13,706	401
**Conf. A**	30	2.0	6350 (−3%)	1983 (+8%)	1521 (+1%)	14,073 (+3%)	400 (−0.2%)
**Conf. B**	30	1.75	6348 (−3%)	1722 (−13%)	1501 (−1%)	13,854 (−2%)	403 (+1%)

**Table 11:** Operating Costs breakdown for configurations under consideration.

	Density (nr./m^2^)	Safety Margin	Cash DOC (US $/h)	Capital Costs (US $/h)	Fuel Costs (US $/h)	Maintenance Costs (US $/h)	Charges (US $/h)	Crew (US $/h)
**Ref.**	0	2.0	3296	3222	935	988	450	1080
**Conf. A**	30	2.0	2945 (−11%)	3405	934	561	469	1080
**Conf. B**	30	1.75	2943 (−11%)	3405	931	459	472	1080

**Table 12:** SHM system characteristics with a lower sensors density.

Component	Density (nr./m^2^)	Estimated Costs (€)	Weight (kg)
Fuselage	10	895,233	319
Wing	10	516,417	184
Horizontal tail	10	128,301	46
Vertical tail	10	104,926	37
**ToT.**		1,644,877	586

**Table 13:** Comparison of weight estimation for Configuration C, designed by imposing a lower value of structural safety margin (SM = 1.75) a lower sensors density (10 per square meter), with respect to reference (without SHM and with SM = 2.0).

	Density (nr./m^2^)	Safety Margin	OEW (kg)	MTOW (kg)	Fuselage (kg)	Wing (kg)	H-Tail (kg)	V-Tail (kg)
**Ref.**	0	2.0	36,189	66,953	7101	6454	812	653
**Conf. C**	10	1.75	36,041 (−0.4%)	65,732 (−2%)	7016 (−1.2%)	6588 (+2%)	811 (−0.1%)	654 (+0.1%)

**Table 14:** Comparison of performance for Configuration C, designed by imposing a lower value of structural safety margin (SM = 1.75) a lower sensors density (10 per square meter), with respect to reference (without SHM and with SM = 2.0).

	Density (nr./m^2^)	Safety Margin	DOC (US $/h)	TO Field Length (m)	Landing Distance (m)	Block Fuel (kg)	Block Time (min)
**Ref.**	0	2.0	6518	1837	1509	13,706	401
**Conf. C**	10	1.75	6209 (−4.7%)	1857 (+1%)	1488 (−1.4%)	13,394 (−2%)	400 (0.1%)

**Table 15:** Operating Costs breakdown for configuration C (compared with all configurations analyzed).

	Density (nr./m^2^)	Safety Margin	Cash DOC (US $/h)	Capital Costs (US $/h)	Fuel Costs (US$/h)	Maintenance Costs (US $/h)	Charges (US $/h)	Crew (US $/h)
**Ref.**	0	2.0	3296	3222	935	988	450	1080
**Conf. A**	30	2.0	2945 (−10.7%)	3405	934	561	469	1080
**Conf. B**	30	1.75	2943 (−10.7%)	3405	931	459	472	1080
**Conf. C**	10	1.75	2926 (−11.2%)	3283	917	458	471	1080

## Data Availability

The data presented in this study are available on request from the corresponding author.
